# Prediction of Autogenous Shrinkage of Concrete Incorporating Super Absorbent Polymer and Waste Materials through Individual and Ensemble Machine Learning Approaches

**DOI:** 10.3390/ma15217412

**Published:** 2022-10-22

**Authors:** Hisham Jahangir Qureshi, Muhammad Umair Saleem, Muhammad Faisal Javed, Abdulrahman Fahad Al Fuhaid, Jawad Ahmad, Muhammad Nasir Amin, Kaffayatullah Khan, Fahid Aslam, Md Arifuzzaman

**Affiliations:** 1 Department of Civil and Environmental Engineering, College of Engineering, King Faisal University, Al-Ahsa 31982, Saudi Arabia; 2 Service Stream Limited Co., Ltd., Chatswood, NSW 2067, Australia; 3 Department of Civil Engineering, COMSATS Institute of Information Technology, Abbottabad 22060, Pakistan; 4 Department of Civil Engineering, Swedish College of Engineering, Wah Cantt 47070, Pakistan; 5 Department of Civil Engineering, Prince Sattam Bin Abdulaziz University, Al-Kharj 11942, Saudi Arabia

**Keywords:** concrete, materials, autogenous shrinkage, super absorbent Polymer, machine learning approaches, ensemble models, validation analysis, statistical measures

## Abstract

The use of superabsorbent polymers, sometimes known as SAP, is a tremendously efficacious method for reducing the amount of autogenous shrinkage (AS) that occurs in high-performance concrete. This study utilizes support vector regression (SVR) as a standalone machine-learning algorithm (MLA) which is then ensemble with boosting and bagging approaches to reduce the bias and overfitting issues. In addition, these ensemble methods are optimized with twenty sub-models with varying the n^th^ estimators to achieve a robust R^2^. Moreover, modified bagging as random forest regression (RFR) is also employed to predict the AS of concrete containing supplementary cementitious materials (SCMs) and SAP. The data for modeling of AS includes water to cement ratio (W/C), water to binder ratio (W/B), cement, silica fume, fly ash, slag, the filer, metakaolin, super absorbent polymer, superplasticizer, super absorbent polymer size, curing time, and super absorbent polymer water intake. Statistical and k-fold validation is used to verify the validation of the data using MAE and RMSE. Furthermore, SHAPLEY analysis is performed on the variables to show the influential parameters. The SVM with AdaBoost and modified bagging (RF) illustrates strong models by delivering R^2^ of approximately 0.95 and 0.98, respectively, as compared to individual SVR models. An enhancement of 67% and 63% in the RF model, while in the case of SVR with AdaBoost, it was 47% and 36%, in RMSE and MAE of both models, respectively, when compared with the standalone SVR model. Thus, the impact of a strong learner can upsurge the efficiency of the model.

## 1. Introduction

Concrete is an extensively used material in the construction domain due to the low cost, high strength, and local accessibility of its components [1,2,3]. Moreover, cementitious materials have been created in a diverse variety, including self-compacting concrete (SCC) [4,5,6], high-performance concrete (HPC) [7,8,9], lightweight concrete (LWC) [10,11,12], and ultra-high-performance concrete (UHPC) [13,14,15]. These materials are chosen carefully based on the requisite mechanical properties as well as required durability and might be vulnerable to a variety of degradations in which the most detrimental effect is cracking in the matrix [16,17,18]. The appearance of these cracks can affect the lifespan of a structure and occurs due to various reasons [17]. One cause of cracking in the cementitious matrix is restrained shrinkage, which occurs during the shrinkage of structural elements. However, it is unable to shrink because of surrounding elements and thus this is the most common within the initial days after casting [18]. Additionally, it is reported that the effect of several shrinkage mechanisms contributes to total shrinkage in concrete including autogenous shrinkage, chemical shrinkage, carbonation shrinkage, drying shrinkage, plastic shrinkage, and thermal shrinkage [16,19,20,21]. Furthermore, the effect of autogenous shrinkage has a major influence on HPC and UHPC during the initial days after casting [22,23,24]. These cementitious materials have adequate durability and possess superior mechanical properties to conventional concrete but have lesser demand for water-cement ratio [23]. Due to the limited quantity of water at hand for cement hydration, as relative humidity declines, self-desiccation progresses rapidly inside the capillary pores resulting in capillary depressions in the cementitious matrix [21,25]. Autogenous shrinkage that ensues under isothermal conditions is stated as the external macroscopic volume reduction is observed as a result of these macroscopic evolutions, and cracking may develop during the initial days/weeks [26,27,28].

The addition of supplementary raw materials inside the matrix of concrete has a significant impact on autogenous shrinkage, and the strength of cementitious composite necessitates a thorough understanding of their relative roles in shrinkage [29,30,31]. It was discovered that in addition to the water-to-binder ratio in concrete, cement fineness has a substantial impact and thus leads to excessive autogenous shrinkage [30]. However, a higher binder-to-aggregate ratio minimizes the autogenous shrinkage due to the restraining result of aggregate. Moreover, it is reported that the addition of silica fume in a high-performance cementitious matrix significantly affects the mechanism of autogenous shrinkage and thus there is a need for its proper monitoring in these composite matrixes [29]. Moreover, the intrusion of 5–10% of silica fume in the matrix significantly increases the mechanism of autogenous shrinkage [20]. This is governed mainly due to the following reasons: (i) refined pore structure, (ii) improved CSH synthesis with a porous structure due to portlandite consumption, and (iii) accelerated hydration and water adsorption around silica fume particles. Furthermore, the intrusion of slag by a 30–50% replacement ratio produces a malignant effect on the shrinkage mechanism as the addition of slag affects the autogenous shrinkage due to enhanced chemical shrinkage [32,33]. Whereas it was observed to produce relative expansion in another experiment and particularly in a few UHPC mixtures. Similarly, the addition of fly ash as partial replacement in cement matrix with a ratio ranging from 15 to 60% can significantly reduce shrinkage due to the slower rate of hydration [34,35]. Additionally, it was shown that calcined clay can decrease short-term autogenous shrinkage but its addition to the composite matrix have a significant effect and thus increases the autogenous shrinkage over a long duration [36,37,38], whereas filler materials are often stated to reduce shrinkage by serving as small aggregate and thus decrease the shrinking of the cement paste [39,40,41]. It is because of these negative effects that it is vital to investigate the autogenous shrinkage behavior of composite concrete formations, particularly in limestone, slag filler mixtures, HPC, and in making environmentally friendly UHPC [37,39,40].

The use of specific additives in concrete results in the mitigation of autogenous shrinkage [26]. From ordinary components to engineered substances, various additives have been utilized. Similarly, the intrusion of lightweight aggregates (pumice) in composite minimizes the mechanism of shrinkage during the initial days after casting because of their inherent porosity allowing water to gradually escape [42]. Recently, the development of superabsorbent polymers (SAP) can considerably minimize concrete shrinkage due to their capability of storing excess water during mixing and releasing it within the first few days [43,44,45]. Over the last few years, SAP has been effectively used to reduce concrete shrinkage in high pH concrete matrixes by optimizing their release rate and water absorption capacity. The use of SAP in the concrete mixture in a percentage of approximately 0.2–0.6% by cement mass has proven to be effective in reducing drying shrinkage, autogenous shrinkage, and stress improvement [17,46,47]. However, some subsequent lateral deformations may be detected when SAP is depleted of its contents. Moreover, their efficiency depends upon their nature, initial cross-linking, and chemical components [17]. Furthermore, it can be measured before mixing concrete by conducting absorption tests through the filtration method and tea bag method. Despite being no accurate chemical composition regarding SAP but having varied compositions and absorption characteristics shows efficacious in alleviating autogenous shrinkage. The diameter of the SAP particles—first thought to be critical in shrinkage reducing capacity—turns out to be a less substantial characteristic as long as the particles are dispersed uniformly in the cementitious matrix, and the intrusion of SAP in the matrix depicts benefits in the mitigation of plastic and drying shrinkage as they increase self-healing and resistance to freeze-thaw [48,49,50,51].

Cracking in contemporary concrete has the potential to cause significant damage, thus it is crucial to know how SAP affects the autogenous shrinkage of mixes including cementitious ingredients in high-performance and ultra-high-performance concrete. Thus, the use of machine learning (ML) anticipation would assist while designing these kinds of complex materials [52,53,54,55,56,57,58]. Many Civil Engineering issues, including concrete strength prediction [59,60,61], creep prediction [62], crack evaluation, foam concrete strength [63,64,65], microstructural features, such as surface chloride content and mechanical behavior of stabilized soil, have been effectively applied to artificial intelligence systems [66,67]. In addition, Table 1 represents applications of MLA in the civil engineering domain to anticipate their desired properties.

This study utilizes the machine learning approaches for the prediction of autogenous shrinkage of the concrete incorporating waste materials SCMs and super absorbent polymer (SAP). A vast set of variables was gathered from published literature with approximately 1889 data points at different days. Supervised algorithm support vector regression (SVR) is utilized as a standalone approach. This approach was then improved by employing a strong learner method namely bagging and boosting to depict its significance. Furthermore, SHAPLEY analysis is performed to check the importance of the parameters. Moreover, random forest (RF) as modified bagging is applied and a comparison is made to show the most influential model in the prediction of autogenous shrinkage of concrete. Furthermore, the individual model is optimized by making twenty models for bagging and boosting to give a robust model R^2^. In addition, statistical measures and validation is used to evaluate the effectiveness of the models.

## 2. Data Description

**Figure 1:**
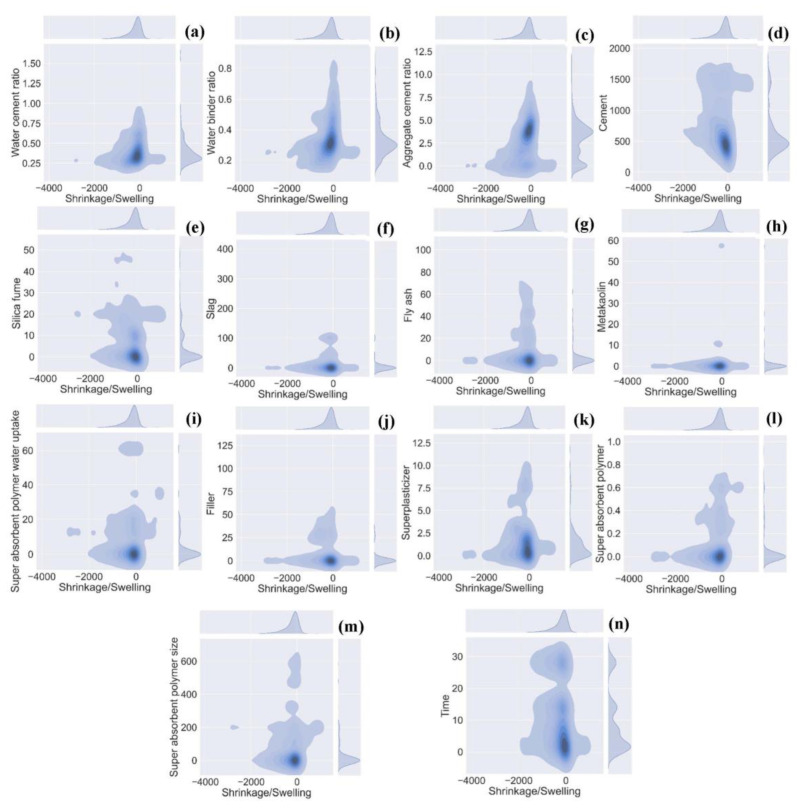
Frequency distribution of input parameters; (**a**) Water cement ratio; (**b**) Water binder ratio; (**c**) Aggregate cement ratio; (**d**) Cement (kg/m^3^); (**e**) Silica fume (% cement mass); (**f**) Slag (% cement mass); (**g**) Flyash (% cement mass); (**h**) Metakaolin (% cement mass); (**i**) Super absorbent polymer water intake g/g of SAP; (**j**) Filler (% cement mass); (**k**) Superplasticizer (% cement mass); (**l**) Super absorbent polymer (% cement mass); (**m**) Super absorbent polymer size µm; (**n**) Curing time (days).

The database required for the prediction of autogenous shrinkage was accumulated from published literature and the NU database, incorporating supplementary cementitious materials and SAP [34,100,101,102,103,104,105,106,107,108,109,110,111,112,113,114,115,116,117,118,119,120,121,122,123,124,125,126,127,128,129,130,131,132,133,134,135,136,137,138,139,140,141,142,143,144,145,146,147,148] (see Supplementary File S1). A total of 1889 data points are extracted on different days ranging from one to twenty-eight days. The variables used in forecasting autogenous shrinkage are comprised of fourteen inputs including water-cement ratio, water binder ratio, aggregate-to-cement ratio, silica fume (% cement mass), cement content (kg/m^3^), slag content (% cement mass), fly ash (kg/m^3^), metakaolin (% cement mass), superplasticizer (% cement mass), filler content (kg/m^3^), SAP content (% cement mass), SAP water uptake (g/g of SAP), SAP size (µm), and curing time (days). Moreover, the RH of the data ranges between 20 to 98%. As the data gathered mostly includes waste materials, thus the main focus is to make a predictive model using the aforementioned variables. The data description and frequency distribution of the variables is shown in Table 2 and Figure 1.

## 3. Machine Learning Methods

**Figure 2:**
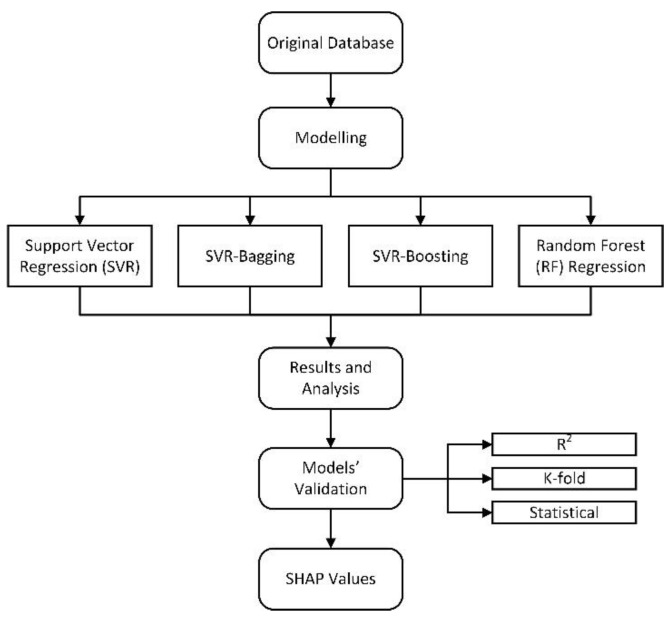
Flow chart of research strategy.

The behavior of the cementitious composite was predicted and evaluated by many researchers using machine-learning algorithms. This research addresses the prediction of autogenous shrinkage of composite matrix incorporating SCM and SAP by deploying artificial intelligence approaches comprising support vector machine (SVM) and random forest (RF). These approaches were selected depending on their high accuracy and popularity in prediction. The SVM provides a very useful technique within it, known as kernel and by the application of the associated kernel function, we can solve any complex problem. Moreover, SVM generally does not suffer the condition of overfitting and performs well when there is a clear indication of separation between classes. The other important advantage of the SVM Algorithm is that it is able to handle High dimensional data too and this proves to be a great help taking into account its usage and application in the Machine learning field. Furthermore, these individual algorithms are used in combination with ensemble bagging and boosting approaches. The overall schematic flow of the method used is depicted in Figure 2.

### 3.1. Support Vector Regression

Support vector regression belongs to a class of supervised machine learning that is used to solve high-dimensional problems. This approach can be used for the classification and regression of data and can also be used for pattern recognition. SVM uses a series of kernel-based functions in order to construct a reliable regression model that forecasts the output values of the prediction models. SVM uses a hyperplane to map a collection of training sets indicating coordinates of points in space-time to a multidimensional feature space. The use of SVM in the modeling of autogenous shrinkage is due to several advantages as it offers handling of high dimensional space data scenarios with more dimensions than specimen counts memory performance. Moreover, it also provides keen flexibility of taking the best kernel function in the prediction of outcome.

### 3.2. Bagging Algorithm (BR) as Ensemble Model

The BR method is an example of a parallel ensemble method. Its purpose is to explain the prediction model’s variance by enhancing it with additive data, while it is in the training stage. This result is derived from an irregular sampling method that uses data substitution from the primary set. Employing replacement sampling techniques makes it possible to repeat specific observations in all-new training data set. During the bagging procedure, the likelihood that each one of the components is included in the newly created dataset is kept constant. When the sizing of the training set is increased, there is no discernible impact on the predictive force. In addition, the deviation can be significantly ablated by adjusting the forecast to more closely match the desirable conclusion. Every one of these data sets is often put to use in the process of training new models. This collection of multiple models takes the average of the predictions made by all models. When using regression, the forecast may be the average or standard of the projections from several various models. Twenty separate models are used to fine-tune the DT in conjunction with the BR in search of the excellent value that will generate a solid output result.

### 3.3. AdaBoost

Boosting is a technique for machine learning that is predicated on constructing a highly accurate prediction rule by compounding various inefficient and incorrect practices. The AdaBoost algorithm developed by Freund and Schapire is the most widely studied and used algorithm today. Its applications can be found in a wide variety of different industries. A supervised machine learning method that operates as part of an ensemble, the AdaBoost regressor can be used to predict future outcomes. Weights are sometimes termed as Adaptive Boosting as they are reallocated to each instance. This is because substantial weights are allotted to cases that were classified incorrectly. Mainly, boosting methods are utilized for supervised learning to minimize variation and bias. These ensemble algorithms are used to improve the performance of weak learners, and they are pretty successful in doing so. When creating the first decision tree or model, high attention is given to recording data that has been improperly categorized. Only this data is passed on as input to the subsequent model. The procedure is carried out numerous times until the desired quantity of base learners has been produced. AdaBoost regressor is considered the most efficient method for improving the performance of decision trees for the classification of binary tasks. It is also possible to use it to enhance the functionality of other machine learning algorithms that are currently being implemented.

### 3.4. Random Forest (RF) Regressor

The term “random forest,” also known as “random decision forests” and “randomised trees”, refers to an ensemble approach to machine learning that uses several decision trees to solve various regression and classification problems (DT). Moreover, a random forest is a collection of different DTs that are all independent of one another. 

The random forest approach has been shown to have strong generalization potential, as showed Breiman [149]. Random forests provide a versatile framework that allows for the selection of objective functions that are task-specific, as well as many categories of separation functions and posterior models. The tree numbers and the depth of the trees in a Random Forest are the two most important hyperparameters. As the number of trees grows, more accurate predictions may be made resulting in a constant reduction in prediction error [66].

### 3.5. Validation of Data with K-Fold

**Figure 3:**
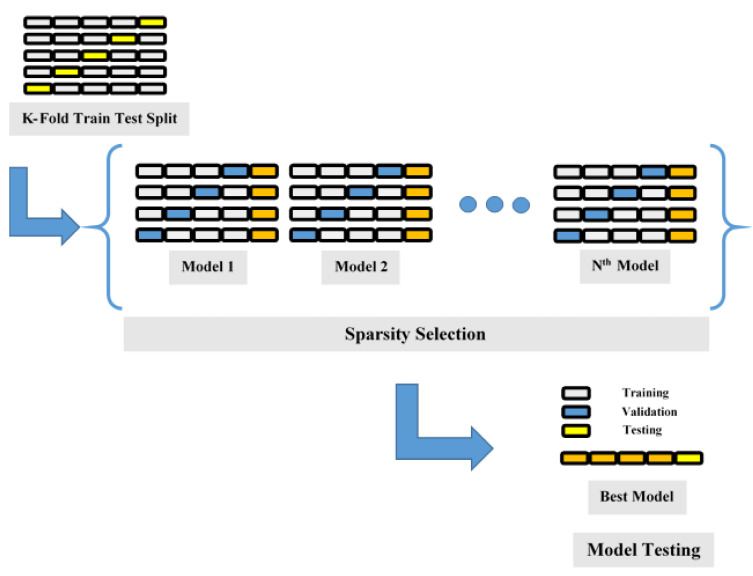
Validation with K-fold cross series.

In most cases, the cross-validation algorithm (k-fold) minimizes the bias associated with random selection, associated with the preparation, and holds out data sampling. According to the results of Kohavi’s research [150], the ten-fold validation test produces a certain deviation, while simultaneously achieving the optimal amount of computational time. To evaluate the model’s performance, this research utilized a method known as stratified ten-fold cross-validation, which divides a predetermined amount of data specimen into ten distinct subdivisions. In each of the 10 phases of model creation and validation, a unique subset of data is used for testing, so that all parts of the model creation process can be independently validated. As seen in Figure 3, the test subset is used to validate the model’s precision. After that, the accuracy of the algorithm is expressed as an average accuracy obtained by ten models throughout all rounds of validation.

### 3.6. Statistical Measures for Model Evaluation

The performance of the predictive model of individual and ensemble learners is evaluated using the mentioned statistical indicators as listed below see Equations (1) and (2) [55].


(1) $$\mathrm{MAE}=\frac{1}{n}\sum_{i=1}^{n}\left|x_{i}-x\right|$$

(2) $$\mathrm{RMSE}=\sqrt{\sum^{\;}\frac{{\left(y_{pred}-y_{ref}\right)}^{2}}{N}}$$

n, N= number of data samples,

$x_{i},y_{pred}=$ predicted data sets,

$x,y_{ref}=$ experimental or reference data sets.

## 4. Result

### 4.1. Support Vector Machine Modeling

**Figure 4:**
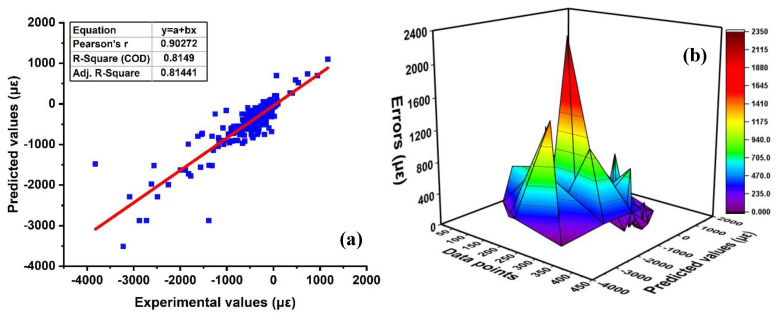
Support vector regression (SVR); (**a**) regression analysis with experimental and predicted result; (**b**) Errors of experimental and targeted via SVR.

The prediction of the autogenous shrinkage using super absorbent polymer via nonlinear regression (SVM) is illustrated in Figure 4. It can be seen that the SVM approach produces results that have a reasonable accuracy and a low degree of variance between the values that were actually measured and those that were predicted. Moreover, the model assessment is evaluated by the coefficient of determination (R^2^) and statistical analysis using MAE and RMSE. The regression analysis as shown in Figure 4a depicts that the model shows robustness performance with R^2^ = 0.81. Similarly, Furqan et al. [67] forecasted the CS of HPC using various MLA and reveals that employing SVM yields R^2^ of approximately 0.81. In addition, Figure 4b represents the error distribution of SVM based model, which includes the distribution of experimental values and projected values. In addition, the distribution of errors in terms of statistical indicators, such as RMSE and MAE of the testing set, shows 241.31 με and 125.082 με, respectively. Moreover, the testing set shows average errors with maximum and a minimum of approximately 125.08 με and 2344.8 με, respectively. In addition, Supplementary File S2 represents the model results of SVM with errors.

#### 4.1.1. Ensemble Modeling Outcome

The evaluation of the non-ensemble model by ensemble algorithm is done by using employing a supervised algorithm using AdaBoost and gradient boosting as discussed below.

#### 4.1.2. AdaBoost Regression 

**Figure 5:**
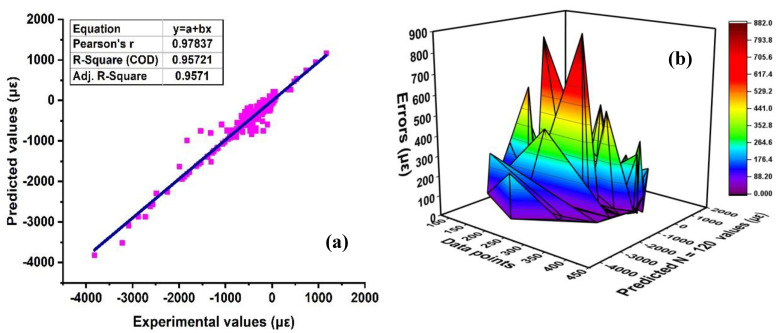
(**a**) SVM with AdaBoost; (**b**) errors of ensemble model of SVM with AdaBoost.

The use of AdaBoost on supervised or non-ensemble SVM algorithms depicts significant and robust performance as compared to the individual SVM as illustrated in Figure 5. This is because the ensemble model uses multiple models to make a strong model with improved results. The ensemble model with the AdaBoost algorithm gives a good response by depicting a higher R^2^ = 0.95 with less error as illustrated in Figure 5a,b. Moreover, the ensemble model enhances the model robustness by 17.28% as compared to SVM individual model. Similarly, the error distribution shows a lesser statistical measure response as demonstrated in Table 3. The error distribution of the ensemble model shows an average error, maximum and minimum errors of approximately 80.40 με and 880.65 με, respectively. This shows that with higher R^2^, the values of statistical errors with RMSE and MAE show 126.75 με and 80.4 με, respectively, as compared to the SVM model. Furthermore, the results of the SVM with AdaBoost are illustrated in Supplementary File S2.

#### 4.1.3. Bagging Regression

**Figure 6:**
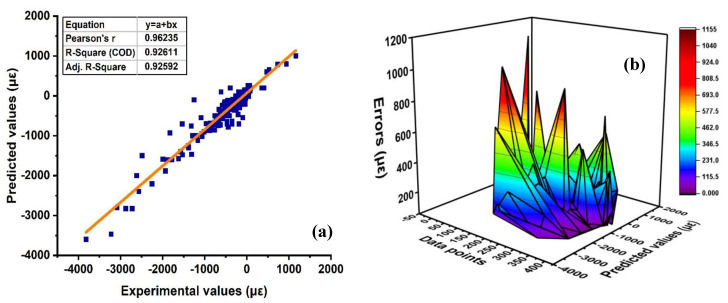
SVM ensemble model; (**a**) SVM with bagging; (**b**) Error distribution of SVM bagging.

The ensemble approach of the SVM model using bagging regression represents a substantial response as compared to the standalone model as illustrated in Figure 6. The response of the experimental results and expected results from the model shows R^2^ = 0.92 as demonstrated in Figure 6a. The model gives a robust performance with less divergence by showing minimal statistical error as compared to the individual one. Thus, the correlative value with R^2^ = 0.92 depicts that the bagging algorithm is way more precise in anticipating the autogenous shrinkage of concrete. Similarly, the distribution of error between anticipated results and experiment values is shown in Figure 6b. In addition, Table 4 represents the distribution of errors in terms of statistical indicators, such as RMSE and MAE of the testing set, showing 183.55 με and 132.49 με, respectively. Moreover, the testing set shows average errors with a maximum and a minimum of approximately 132.49 με and 1151.2 με, respectively. Similarly, Supplementary File S2 depicts the result of the model.

### 4.2. Random Forest Regression

**Figure 7:**
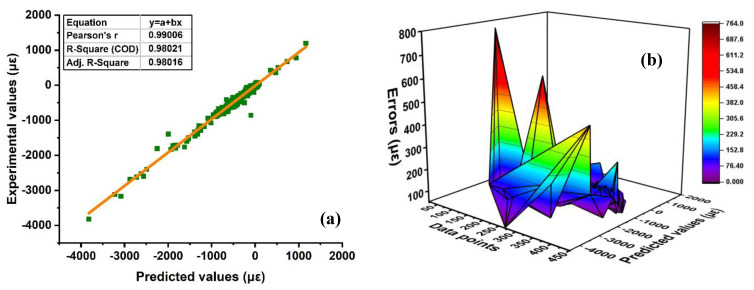
Random forest model; (**a**) regression analysis of experimental and predicted models; (**b**) distribution error of RF model.

The anticipated outcome of the AS of concrete via ensemble supervised random forest is depicted in Figure 7. It shows that the anticipated model results are close to the experimental results with less or minimal errors and thus, shows magnificent R^2^ = 0.98 as illustrated in Figure 7a. Similarly, Furqan et al. [67] predict the strength of concrete using individual and RF approaches, and the same response was observed with maximum R^2^ = 0.96. Moreover, the accuracy of the model can also be illustrated by its distribution of errors and statistical measures. Figure 7b represents the model error distribution with a maximum, minimum, and an average error of approximately 763.25 με and 0.02 με, respectively; the statistics indicates in term of RMSE and of MAE of the testing set for the RF model shows errors of approximately 80.65 με and 46.13 με, respectively. The model results are shown in Supplementary File S2.

## 5. Cross Validation

**Figure 8:**
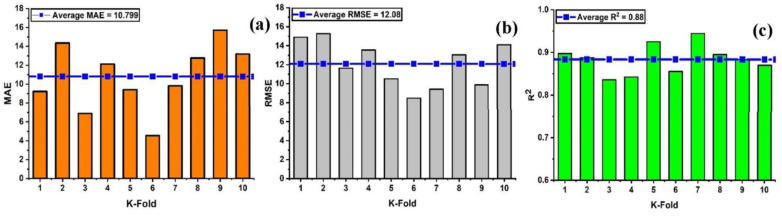
Cross validation; (**a**) SVR with MAE; (**b**) SVR with RMSE; (**c**) SVR with R^2^.

**Figure 9:**
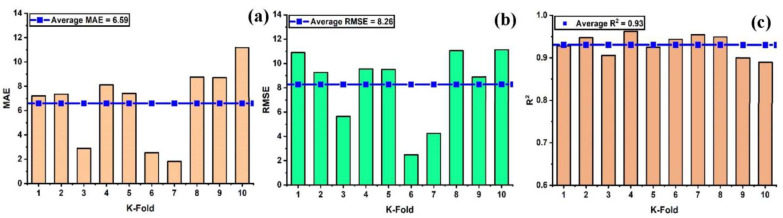
Cross validation; (**a**) SVR-adaboost with MAE; (**b**) SVR-adaboost with RMSE; (**c**) SVR-adaboost with R^2^.

**Figure 10:**
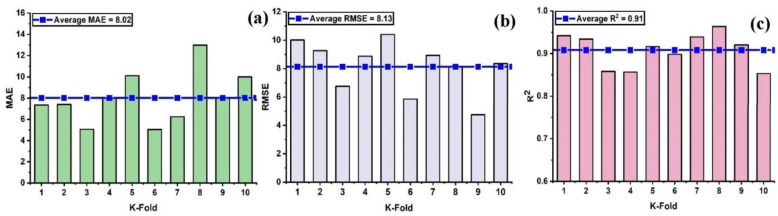
Cross validation; (**a**) SVR-bagging with MAE; (**b**) SVR- bagging with RMSE; (**c**) SVR-bagging with R^2^.

**Figure 11:**
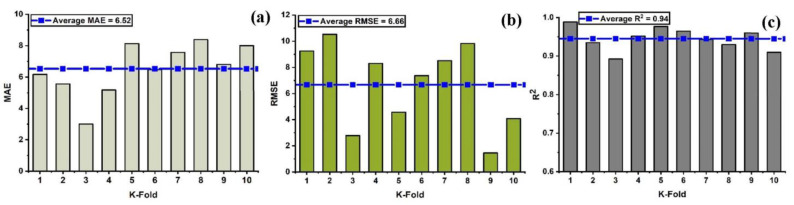
Cross-validation; (**a**) RF with MAE; (**b**) RF with RMSE; (**c**) RF with R^2^.

In order to properly evaluate a model’s effectiveness, one must first determine the level of accuracy they require. Thus, validation is necessary for this purpose in order to guarantee the correctness of the prediction models. The K-fold validation test is used to verify the correctness of data via shuffled data [59]. This method is utilized to reduce the amount of bias that results from randomly selected samples from the training data set [60]. It does it by dividing the observations of the experimental results into ten equal sections and makes use of nine out of ten subsets in order to give the robust learner. Moreover, the tenth subset is the one that is used in order to validate the model [52]. Moreover, this procedure is carried out a total of 10 times after which an accurate measurement that is representative of all ten runs is acquired. In general, it’s commonly accepted that the 10-fold cross-validation approach accurately reflects the model’s generalizability and dependability [150]. Figure 8 illustrates the validation test that was performed on the nonlinear model (SVM) with some statistical measures as illustrated in Figure 8a–c. Similarly, all of the models exhibit moderate to high correlation relationships that range from moderate to strong as depicted in Figure 9a–c and Figure 10a–c. In addition, the outcomes of the cross-validation process further are examined for RFR from the perspective of various errors, such as the root mean square error and mean absolute error. It can be seen that the variations have been observed in the statistical measures but yet, the degree of accuracy has remained rather high as depicted in Figure 11a–c. Moreover, their validation results are also shown in Table 5 and Table 6.

## 6. Discussion on Assessment of Models via Statistical Indicators

Comparisons were made between each other to demonstrate better the capabilities of the ensemble algorithm concerning the different individual machine learning algorithms. The determinations of both the ensemble model and model parameters are very similar. All the values that are validated and targeted are displayed in Figure 4, Figure 5, Figure 6, Figure 7, Figure 8, Figure 9, Figure 10 and Figure 11. This unequivocally demonstrates that results obtained from ensemble machine learning models exhibit a linear pattern and predictions obtained from these models are significantly closer to the actual values. This is due to the reason that SVR is considered an individual learning approach, whereas bagging and boosting with MLA are deemed considered as ensemble modeling. Weak learners that have demonstrated above-average performance will see their weight increase, while weak learners that have demonstrated below-average performance will see their weight decrease. It is often known that ensemble learning involves many vulnerable learners generated by individual learning algorithms. Compared to the ensemble learner with bagging and boosting, the error values produced by individual learners are significantly higher. This demonstrates that not only do the ensemble models have accurate predictions, but they also help reduce the error range that exists between those targets and predictions.

## 7. Feature Importance Analysis Using Shapley Additive Explanations (SHAP)

**Figure 12:**
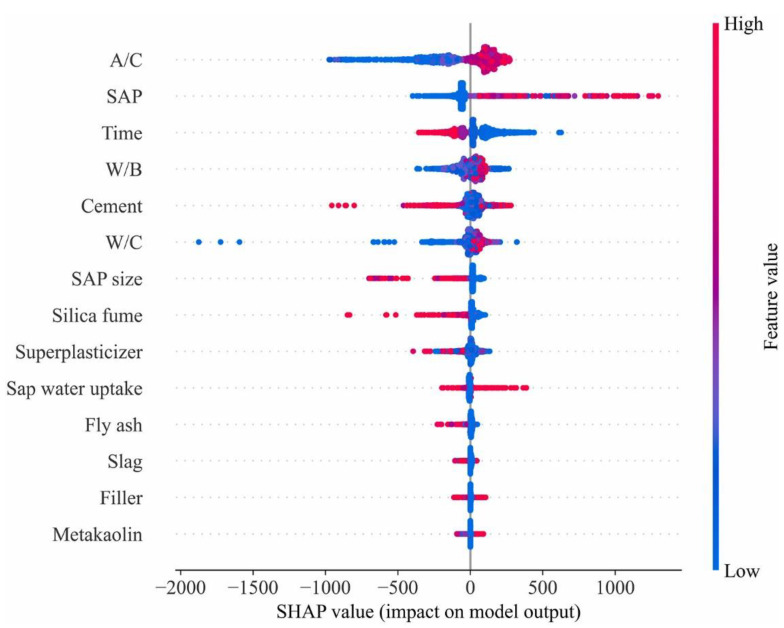
Feature importance analysis using the SHAP library in Python code.

The Shapley Additive explanation (SHAP) gives both internal and external explanations of every input variable in this investigation. SHAP delivers equivalent information to the widely used feature significance metric but is better suitable for collective machine learning techniques since it is more robust and gives quantitative and qualitative information. Figure 12 displays the SHAP scores of each attribute, ordered by their mean SHAP value. The characteristics shown at the top of the pictorial depiction are connected with the largest model output contributions. The characteristics that influenced shrinkage forecasts the most were the aggregate-to-cement ratio (A/C), the SAP content, time (days since the commencement of shrinkage measurements), water-to-binder ratio, amount of cement, water-to-cement ratio, SAP size, and silica fume concentration. For each of these parameters, there is a distinct dividing line between high and low effects on model output: high A/C ratio increases SHAP value, which decreases shrinkage; high SAP content decreases shrinkage and was discovered to be the greatest important variable; high time values necessarily correlate to higher shrinkage values; high w/b and w/c tend to decrease shrinkage, whereas high silica fume replacement ratio or cement content generally induces higher shrinkage, and big SAP size reduces SAP positive effect and enhances shrinkage compared to smaller SA. These impacts are consistent with the results of experiments. It was determined that superplasticizer, fly ash, slag, filler, and calcined clay content had the least influence. These SHAP-based results are compatible with the experimental measurements, as these factors are known to have a negligible effect on shrinkage.

## 8. Limitations and Future Work

Despite the fact that the work given in this study has significant limitations, it may still be regarded as data mining-based research. Completeness of data is essential for the efficacy of prediction models. The data ranges used in this research were limited to 1889 points. In addition, the tensile and corrosive behavior of concrete at extreme temperatures was not considered in this work. Indeed, good database management and testing are essential from a technical standpoint. Nonetheless, this investigation has a vast array of datasets containing variables for the modeling of high-strength concrete. Further, it is suggested that a new set of data concrete at increased temperatures that encompasses numerous environmental factors, such as heat, rust, and longevity, be investigated. As concrete plays a crucial part in the environment, its effects under various situations should be investigated by utilizing various deep machine learning methods, such as convolutional neural networks (CNNs), recurrent neural networks, and limited Boltzmann machines (RBM).

## 9. Conclusions

This study utilizes the supervised machine learning algorithms in predicting the response of autogenous concrete having super absorbent polymer and waste materials with 1889 datasets. The results are in accordance with the literature with improved outcomes for models used in this study. These models then ensemble with strong learners. The following conclusions are drawn as listed below.

1. The utilization of machine learning with bagging and boosting on individual methods depicts a strong relation in making models by employing huge data sets;
2. Ensemble learners and modified bagging illustrate a strong link between target and experimental results as compared to individual learners;
3. Boosting with AdaBoost and modified bagging with random forest on the SVR model offers robust performance with R^2^ of approximately 0.95 and 0.98, respectively. The ensemble model illustrates 17% and 21% enhancement as compared to the individual SVR model with an R^2^ of 0.81;
4. The efficiency of the model is also assessed by computing RMSE and MAE as statistical indicators. It is observed that modified bagging illustrates 67% and 63% enhancement for RMSE and MAE as compared to the SVR model, respectively. Similarly, SVR with AdaBoost depicts 47% and 36% enhancement with the same statistical parameters. Thus, both models give a strong performance as compared to the standalone method;
5. Authentication of data via cross-validation with statistical measures using MAE, RMSE, and R^2^ was done. The model illustrates vigorous robust performance with fewer errors.

This work will pave the way for future research into the appropriate use of SAP in cement-based materials, including SCM. For instance, the insights can affect the decision of concrete constituents to reduce autogenous shrinkage during a short period. It is suggested that additions be made to the database so that it may be expanded collaboratively, by incorporating chemical formulations of SAP or shrinkage measuring methodologies. Coupled with estimates of mechanical characteristics, it is also possible to foresee possible future developments. Such sophisticated models may be of relevance in the future for the production of sustainable and environment-friendly cementitious materials with ultra-high performance.

## Figures and Tables

**Table 1:** MLA in civil engineering domain.

Sr. No	Machine Learning Method	Prediction Property	Abbreviation	Waste Materials	Data Set	Year	References
1.	Gene Expression Programming	Compressive strength	GEP	Super plasticizers	357	2020	[68]
2.	Support Vector Machine	Compressive strength	SVM	Normal concrete	15	2021	[69]
3.	Individuals with ensemble modeling	Compressive strength	ANN, bagging and boosting	FA GGBFS	1030	2021	[70]
4.	Gene expression programming	Compressive Strength	GEP	GGBFS	351	2020	[71]
5.	Support Vector Machine Adaptive-Network-based Fuzzy Inference System	Deflection	SVM-ANFIS	RC beam	120	2020	[72]
6.	Support vector machine	Slump test, L-box test, V-funnel test, Compressive strength	SVM	FA	115	2020	[73]
7.	Gene expression programming	Compressive Strength	GEP	FA	298	2021	[74]
8.	Adaptive neuro-fuzzy inference system	Compressive strength	ANFIS with ANN	POFA	7	2020	[75]
9.	Gene expression programming and random forest	Compressive strength	GEP and RF	-	357	2020	[76]
10.	Multivariate	Compressive strength	MV	Crumb rubber with SF	21	2020	[77]
11.	Artificial neuron-network	Compressive strength	ANN	FA GGBFS SF RHA	205	2019	[78]
12.	Gene expression programming	Post fire behavior	GEP	GGBFS	160	2020	[79]
13.	Intelligent rule-based enhanced multiclass support vector machine and fuzzy rules	Compressive strength	IREMSVM-FR with RSM	FA	114	2019	[80]
14.	Data Envelopment Analysis	Compressive strength, Slump test, L-box test, V-funnel test	DEA	FA	114	2021	[81]
15.	Random forest	Compressive strength	RF	FA GGBFS SF	131	2019	[82]
16.	Support vector machine	Compressive strength	SVM	FA	-	2020	[83]
17.	Artificial neuron-network	Compressive strength	ANN	FA	114	2017	[84]
18.	Conventional Artificial-Neural Network	Compressive Strength	C-ANN	Foamed concrete	220	2020	[85]
19.	Ensemble models	Unconfined compressive strength	RT, RF, GBRT, ensemble GBRT	Cemented Paste Backfill	126	2019	[86]
20.	Gene expression programming	Tensile Strength	GEP	Normal concrete	168	2012	[87]
21.	Artificial neuron-network	Compressive strength	ANN	FA	300	2009	[88]
22.	Multivariate adaptive regression spline	Compressive strength Slump test L-box test V-funnel test	M5 MARS	FA	114	2018	[89]
23.	Artificial Neural Network Multi Linear Regression	Compressive strength	ANN and MLR	Clinker mortar	1288	2015	[90]
24.	Gene expression programming	Axial capacity	GEP	-	277	2020	[91]
25.	Artificial neuron-network	Compressive strength	ANN	FA	69	2017	[92]
26.	Artificial neuron-network	Compressive strength	ANN	FA	80	2011	[93]
27.	Biogeographical-based programming	Elastic modulus	BBP	SF FA SLAG	413	2016	[94]
28.	Artificial Neural-Network	Thermal properties	ANN	Silica fume	264	2019	[95]
29.	Support Vector Machine Random forest AdaBoost	Compressive Strength	SVMRFAB	Blast furnance slag and waste tire rubber powder	288	2017	[96]
30.	Adaptive neuro-fuzzy inference system	Compressive strength	ANFIS	-	55	2018	[97]
31.	Artificial neuron-network	Compressive strength	ANN	FA GGBFS SF RHA	169	2016	[98]
32.	Random Kitchen Sink Algorithm	V-funnel test J-ring test Slump test Compressive strength	RKSA	FA	40	2018	[99]

**Table 2:** Parameters description used in modeling.

Parameters	Abbreviations	Units	Maximum	Minimum	Median	Average
Water cement ratio	W/C	-	1.6	0.17	0.35	0.39
Water binder ratio	W/B	-	0.86	0.157	0.3	0.33
Aggregate to cement ratio	AA/C	-	11.56	0	3.28	2.82
Cement	C	kg/m^3^	1762	167.4	498	637.40
Silica fume	SF	(% cement mass)	50	0	0	4.80
Filler	F	(% cement mass)	100	0	0	5.12
Flyash	FA	(% cement mass)	400	0	0	8.05
Slag	SL	(% cement mass)	57.4	0	0	0.70
Metakaolin	MK	(% cement mass)	125	0	0	4.51
Superplasticizer	SP	(% cement mass)	11.82	0	0.8	1.39
Super absorbent polymer	SAP	(% cement mass)	0.92	0	0	0.06
Super absorbent polymer size	SAPS	µm	645	0	0	43.00
Super absorbent polymer water	SAPW	g/g of SAP	61	0	0	4.50
Curing time	T	days	28	0	7	9.06
Output						
Shrinkage/Swelling	SH	µε	1166.7	−3818.9	−136.6	−280.92

**Table 3:** SVM model statistical measures to depict its significance.

Statistical Measures	SVM Model	AdaBoost Ensemble Model
R^2^	0.81	0.957
RMSE	241.31	126.34
MAE	125.08	80.4

**Table 4:** Statistical analysis of the SVM model with bagging.

Statistical Measures	SVM Model	Bagging Ensemble Model
R^2^	0.81	0.92
RMSE	241.31	183.55
MAE	125.08	132.49

**Table 5:** Cross-validation results of the SVM model with ensemble algorithms.

Models	SVR			SVR-Adaboost			SVR-Bagging		
K-Fold	MAE	RMSE	R^2^	MAE	RMSE	R^2^	MAE	RMSE	R^2^
1	9.21	14.90	0.90	7.21	10.90	0.93	7.35	10.01	0.94
2	14.35	15.27	0.89	7.35	9.27	0.95	7.41	9.26	0.93
3	6.89	11.64	0.84	2.89	5.64	0.91	5.07	6.75	0.86
4	12.11	13.55	0.84	8.11	9.55	0.96	7.97	8.87	0.86
5	9.41	10.51	0.92	7.41	9.51	0.92	10.11	10.40	0.92
6	4.53	8.48	0.86	2.53	2.48	0.94	5.03	5.86	0.90
7	9.82	9.42	0.94	1.82	4.25	0.95	6.24	8.93	0.94
8	12.76	13.05	0.89	8.76	11.05	0.95	12.99	8.15	0.96
9	15.71	9.88	0.88	8.71	8.88	0.90	8.05	4.76	0.92
10	13.19	14.13	0.87	11.19	11.13	0.89	9.99	8.36	0.85

**Table 6:** Cross-validation results of the RFR model with statistical measures.

Model	RFR		
K-Fold	MAE	RMSE	R^2^
1	6.17	9.25	0.99
2	5.56	10.53	0.93
3	3.01	2.78	0.89
4	5.18	8.31	0.95
5	8.13	4.57	0.98
6	6.47	7.37	0.96
7	7.57	8.52	0.94
8	8.39	9.83	0.93
9	6.80	1.45	0.96
10	8.01	4.08	0.91

## Data Availability

See Supplementary file.

## Supplementary Materials

The following supporting information can be downloaded at: https://www.mdpi.com/article/10.3390/ma15217412/s1.
